# Effect of Oral Vasopressors Used for Liberation from Intravenous Vasopressors in Intensive Care Unit Patients Recovering from Spinal Shock: A Randomized Controlled Trial

**DOI:** 10.1155/2022/6448504

**Published:** 2022-01-18

**Authors:** Ahmed Talaat Ahmed Ali, Mervat Anwar Abd EL-Aziz, Ahmed Mohamed Abdelhafez, Amr Mohamed Ahmed Thabet

**Affiliations:** ^1^Anesthesia and Intensive Care Department, Faculty of Medicine, Assiut University, Assiut, Egypt; ^2^Critical Care and Emergency Nursing Department, Faculty of Nursing, Assiut University, Assiut, Egypt

## Abstract

**Background:**

Early vasopressor utilization has been associated with improved outcomes of patients with spinal shock; however, there are difficulties in weaning off vasopressors, in which patients after recovery from spinal shock develop a state of persistent vasodilation, which may take a few days to resolve and delays the discharge in the intensive care unit (ICU). Therefore, we tested the hypothesis using two oral vasopressors (midodrine and minirin) to facilitate weaning off intravenous vasopressors, reducing the ICU length of stay, and compare them for more efficacy.

**Methods:**

A randomized controlled trial was conducted in the trauma ICU at the Assiut University Hospital in Egypt in patients with spinal shock who required intravenous vasopressor for ≥24 h. A convenience sample was classified into three groups, in which 30 patients were included for each group. The midodrine group received midodrine 10 mg per oral every 8 h with gradual weaning off intravenous (IV) vasopressor (noradrenaline) after receiving 4 doses, the minirin group received minirin 60 *μ*g per oral every 8 h with gradual weaning off IV vasopressor after receiving 4 doses, whereas the control group received IV vasopressor (noradrenaline) with gradual weaning according to the routine hospital care without adding oral vasopressors. The primary outcome was shortening the duration of IV vasopressor requirements. The secondary outcome was reducing the ICU length of stay.

**Results:**

Our results showed that the duration of IV vasopressor requirements in the midodrine (3.3 ± 1.32) and minirin groups (4.8 ± 1.83) was significantly lower than in the control group (6.93 ± 2.32). Additionally, the ICU length of stay (days) in the midodrine (5.13 ± 1.83) and minirin groups (5.5 ± 1.91) was significantly lower than in the control group (9.03 ± 3.74).

**Conclusion:**

Midodrine and minirin accelerated liberation from intravenous noradrenaline and effective in reducing the ICU length of stay in patients with spinal shock.

## 1. Introduction

Spinal shock is a result of severe spinal cord injury. It usually requires high-impact, direct trauma that leads to spinal cord injury and spinal shock [1]. Spinal shock has been described initially in a patient with a transected spinal cord and difficult to treat hypotension as a result of decreased sympathetic tone throughout the body and especially in the arterial wall [1].

In spinal shock, there is a transient increase in blood pressure due to the release of catecholamines. This is followed by a state of hypotension, flaccid paralysis, urinary retention, and fecal incontinence. The symptoms of spinal shock may last a few hours to several days/week [1]. Managing spinal shock is challenging, and there are many guidelines used to keep mean arterial pressure (MAP) above 85–90 mmHg to maintain cord perfusion and reduce ischemia/secondary injury, whereas early vasopressor utilization has been associated with improved outcomes [2].

Eventually, within a few days, hypotension improves, and intravenous (IV) drips should be gradually decreased. However, there are difficulties in weaning off vasopressors, in which patients after recovery from spinal shock develop a state of persistent vasodilation (vasoplegia), which may take a few days to resolve. This delays the discharge in the intensive care unit (ICU) due to a persistent requirement for low-dose intravenous vasopressors [3]. At this point, low doses of IV vasopressors are required for several days. Whenever the vasopressor is stopped, the MAP falls into the mid-50s, which leads to the search for the best approach to manage this [3].

The first approach is bolus with fluid as fluid resuscitation is an inappropriate intervention to aid in weaning off vasopressors since patients' likelihood of volume depletion is essential after being admitted to the ICU for many days [4]. The second approach is to perform bedside ultrasonography and administer fluid if the intravascular capacity (IVC) has lots of respiratory variability and then try to wean off vasopressors. Ultrasound-guided fluid resuscitation may be helpful during the initial resuscitation, but not on ICU day 3 [4]. The third approach is weaning off vasopressors by measuring urine output; this is not a bad approach, but it is not perfect either. Even if the kidneys continue to produce urine, there is still a possibility that they could become injured due to hypotension [4].

A new approach is to wean off vasopressors by using oral vasopressors, such as midodrine or desmopressin, in appropriately selected patients with careful monitoring. This may reduce the ICU length of stay and avoid ICU complications, such as central line infection and delirium. Fast transition to the ICU could facilitate great mobility and avoid deconditioning [5].

Midodrine is an oral agent which functions as an alpha-1 agonist. It has been used in a variety of situations, including autonomic dysfunction, hepatorenal syndrome, and dialysis-induced hypotension. Over the past few years, there has been an increasing interest in using midodrine to facilitate weaning off vasopressors [5]. Oral desmopressin acetate (minirin) is a vasopressin analogue of the natural pituitary hormone 8-arginine vasopressin, antidiuretic hormone (ADH) affecting renal water conservation and causing elevation in blood pressure [6].

In this randomized controlled study, we aimed to evaluate the effect of the use of oral vasopressors (midodrine or minirin) on patients who are in the ICU weaning off IV vasopressors and compare the efficacy in shortening the duration of IV vasopressor requirements and ICU length of stay (LOS).

## 2. Materials and Methods

A randomized controlled study conducted in the trauma ICU at the Assiut University Hospital in Egypt was reviewed and approved by the Medical Ethics Committee in the Faculty of Medicine in Assiut University in Egypt (IRB no: 17300491) and performed in accordance with the ethical standards of the Declaration of Helsinki. Written informed consent was obtained from all patients or their legally authorized representatives prior to their inclusion in the study and after they had been informed of the benefits and risks of the investigation. We checked their electronic medical records to determine the eligibility criteria for the study. The study is registered in the ClinicalTrials.gov/NCT04586790. This study adheres to the CONSORT guidelines.

Data were gathered between October 2020 and April 2021. The shortening of the duration of IV vasopressor requirements was considered the primary outcome, whereas the secondary outcome was reducing the ICU LOS. The inclusion criteria were as follows: patients diagnosed with spinal shock and in the recovery stage, aged 18 years or older, hemodynamically stable, and have stable blood pressure on single agent infusion of noradrenaline (<8 mcg/min). The exclusion criteria were as follows: anuric or oliguric patients or patients with chronic kidney disease and patients with allergy to medications included in the study.

### 2.1. Research Hypothesis

The duration of IV vasopressor requirements and ICU LOS in patients who received oral vasopressors added to IV vasopressors would be significantly shorter than in patients receiving IV vasopressors only.

### 2.2. Sample Size

A power calculation estimated to detect an effect size of 2.5, difference in the mean of the total duration of IV vasopressors between the three studied groups, with a *P* value < 0.05 and 80% power, 0.95 confidence level, and a sample size of 27 patients for each group was needed. However, 60 patients were attempted in this research work to avoid nonresponse rates (30 for each group). This was calculated using the G Power 3.1 [7].

### 2.3. Randomization

Eligible patients were randomized into three equal groups, in which 30 patients were included for each group: control group, midodrine group, and minirin group. Randomization occurred through data generated by the random.org online software. The researchers generated the sequence of numbers “blind” to the study after the selection of patients for eligibility criteria and disclosed prior to the start of the intervention program.

### 2.4. Intervention

Baseline descriptive data collection occurred on the day of enrolment, which includes age, gender, and preexisting comorbidities, such as diabetes, coronary artery disease, asthma, peripheral vascular disease, renal failure, psychiatric disease, musculoskeletal disease, and others, obtained from the patient, patients' family, and patients' medical charts. Baseline laboratory data were recorded, including serum creatinine and arterial blood gas test. The midodrine group received midodrine 10 mg per oral (PO) three times per day (q 8 h) with gradual weaning off IV vasopressor (noradrenaline) after receiving 4 doses, and the minirin group received minirin 60 *μ*g PO three times per day (q 8 h) with gradual weaning off IV vasopressor after receiving 4 doses, whereas the control group received IV vasopressors (noradrenaline on single agent infusion of dose <8 mcg/min) with gradual weaning according to the routine hospital care without adding oral vasopressors.

In the study, hourly monitoring of hemodynamic parameters by 24 h cardiac monitor to perform a continuous recording of mean arterial pressure (MAP), heart rate (HR), respiratory rate (RR), dose of intravenous vasopressors, and assessment of fluid balance was recorded. The main outcomes were measured by time in hours from initiation of oral vasopressors until discontinuation of IV vasopressors was recorded. At the time of the ICU discharge, the following variables were collected: time of ICU discharge and ICU length of stay (LOS). After discharge, follow-up variables rate of ICU readmission, hospital LOS, and any other complications were recorded.

### 2.5. Safety

The patient was kept in the ICU for observation within 24 h following discontinuation of the study drugs to reduce the likelihood of hypotension or other side effects on discharge. If the blood pressure goal was met for more than 24 h without IV vasopressors, the study drug was discontinued prior to discharge to the ward. The accepting team was informed on the discharge that the patient had received a study drug. Instructions were given to the medical and nursing staff to contact a physician investigator if the patient became hypotensive in 24 h after the discharge in the ICU (defined as SBP <90 mmHg).

### 2.6. Statistical Analysis

All analyses were performed using the Statistical Package for the Social Sciences (SPSS) Statistical Software (IBM SPSS Statistics for Windows, Version 21.0. Armonk, NY, USA). Continuous variables were presented as mean ± SD and categorical variables as frequencies. Differences between the groups at baseline were evaluated by an unpaired *t*-test or the Mann–Whitney test for the comparison of continuous variables. The chi-square test or Fisher's exact test was employed to compare categorical variables. Analyses were performed by comparing baseline and postintervention variables in the subgroups (the control group versus the midodrine group or minirin group).

## 3. Results

**Figure 1:**
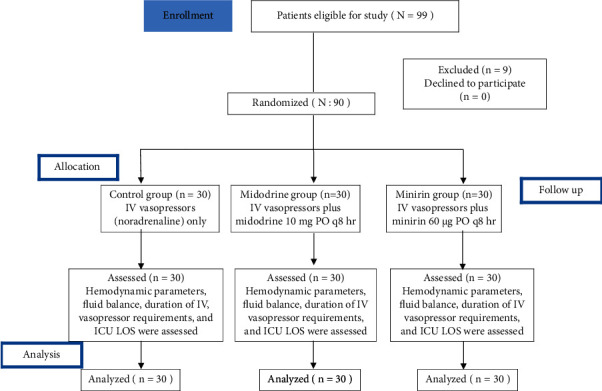
CONSORT flow diagram of a randomized controlled trial.

Ninety-nine patients with spinal shock in the trauma ICU were evaluated according to the eligibility criteria for possible admission to the study, in which 90 patients were included.  Figure 1 shows the flowchart of patient selection and composition of the groups.

**Figure 2:**
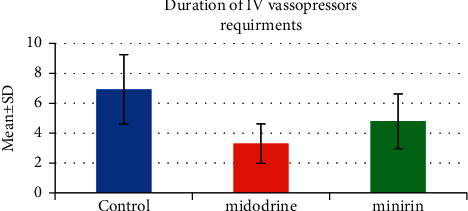
IV vasopressor requirements time.

**Figure 3:**
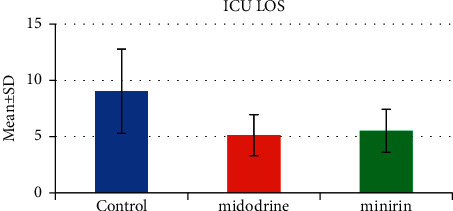
ICU LOS.

The use of oral vasopressors had caused a highly statistically significant decrease in the duration of IV vasopressor requirements in the oral vasopressors group, midodrine (3.3 ± 1.32) and minirin (4.8 ± 1.83), than in the control group (6.93 ± 2.32) (*P*=<0.001) (Table 1 and Figure 2). Moreover, the application of oral vasopressors showed a highly statistically significant decrease in the ICU LOS in the oral vasopressors group, midodrine (5.13 ± 1.83) and minirin (5.5 ± 1.91), when compared to the control group (9.03 ± 3.74) (*P*=<0.001) (Table 1 and Figure 3). Furthermore, there was no significant difference between the midodrine and minirin groups regarding the ICU LOS (*P*3 = 0.592), but it was found that there was a highly statistically significant difference in the duration of IV vasopressor requirements between the two oral vasopressors groups (*P*=0.003), in which the midodrine group greatly reduced the IV vasopressor requirements.

Results show that there are no statistically significant differences between the studied groups regarding the majority items of hemodynamic parameters and laboratory findings except HR in the last period of the study, RR and MAP, and serum creatinine in the first day of the study (*P* value < 0.05), as given in Table 2.

Results show that there are highly statistically significant differences between the studied groups regarding the total intake of fluid in the midperiod and last day of the study (*P*=<0.001). Additionally, there are statistically significant differences between the studied groups regarding the total input in the first and last period of the study (*P* value < 0.05), as given in Table 3.

## 4. Discussion

The current study supports our hypothesis regarding the positive effect of oral vasopressors (midodrine and minirin) in reducing the IV vasopressor requirements and ICU LOS.

The results showed that patients receiving oral vasopressors (midodrine and minirin) had fewer days to be weaned off IV vasopressor (noradrenaline) when compared to the control group who did not receive midodrine or minirin. In the midodrine group, patients took approximately 2–4 days, and the minirin group took approximately 3–7 days to be discontinued in noradrenaline, while the control group took approximately 5–9 days. This was in line with the study by Poveromo et al. [8], which suggested that midodrine has potential as a useful adjunctive treatment in the weaning off IV vasopressor infusions in difficult to wean patients who are otherwise stable. There is also a case report by O'Donnell and Synnott [9] who reported on the use of midodrine 10 mg three times daily to wean a patient from noradrenaline infusion post C7 to T6 laminectomy for spinal cord compression secondary to a leukemic deposit.

There was a case series performed by Sharma et al. [10], who did a case series of four patients who were treated with oral midodrine in a telemetry unit to prevent the requirement for receiving intravenous vasopressor therapy. All patients were treated with midodrine 10 mg q 8 for 24 h, with excellent results and no complications. This comes in line with a retrospective descriptive study performed by Liu et al. [11], comparing 20 patients with shock who were weaning off IV vasopressors using midodrine versus 20 patients weaned without midodrine. The average duration of the intravenous vasopressor used was 0.3 days shorter in patients receiving midodrine (*P*=0.049) than in minirin. All of these studies support our results.

The current study showed that the ICU LOS in the midodrine (3–7 days) and minirin groups (4–8 days) was shorter than in the control group (4–13 days). This comes in line with the study performed by Cardenas-Garcia et al. [12], who reported that there was a reduction in the mean IV vasopressor duration (2.9 days versus 3.8 days, *P* < 0.001) and the ICU length of stay (7.5 versus 9.4 days, *P*=0.017) in the IV vasopressor plus the midodrine group. However, comes in inferior with the study performed by Whitson et al. [13], which revealed that the ICU LOS in patients who received IV vasopressors with midodrine was 7.5 ± 5.9 days versus 9.4 ± 6.7 days in patients who received IV vasopressors only. A study by Santer et al. [14] reported that there was no effect of adding midodrine to IV vasopressors in reducing the ICU LOS.

The current study revealed that there were no differences in the majority of items of hemodynamic parameters between the studied groups except HR in the last period of the study and RR and MAP in the first day of the study, which may be related to other factors that can affect in reducing the ICU LOS rather than vasopressors, and safety measures were taken to keep patients in stabilized condition all period of the study. The results showed that HR in the midodrine group is lower than in the minirin and control groups, with the presence of a statistically significant difference between the groups, which may be explained by the effect of midodrine in the activation of the baroreceptor reflex, similar to other *α*1-agonists.

The current study showed that there was a statistically significant difference between the control and midodrine groups compared with the minirin group regarding the total input in the midperiod and last day of the study, which may be related to the antidiuretic effect of minirin (desmopressin acetate) on renal water conservation.

## 5. Conclusions

In conclusion, oral vasopressors (midodrine and minirin) reduced the time in discontinuation of IV vasopressors in patients who are critically ill with spinal shock, which supports the routine use of midodrine and minirin as oral vasopressors to accelerate liberation from IV vasopressors in the ICU. In addition, it can be concluded that midodrine and minirin had a positive effect on shortening the ICU LOS. The study also described that the use of midodrine is better than minirin in decreasing the duration of IV vasopressors requirements.

## Figures and Tables

**Table 1:** Comparison between the three studied groups related to the duration of IV vasopressor requirements and ICU LOS (*n* = 90).

Outcomes	Control	Midodrine	Minirin	*P* value	*P*1	*P*2	*P*3
	Mean ± SD	Mean ± SD	Mean ± SD				
Duration of IV vasopressors requirements (days)	6.93 ± 2.32	3.3 ± 1.32	4.8 ± 1.83	<0.001^*∗∗*^	<0.001^*∗∗*^	<0.001^*∗∗*^	0.003^*∗∗*^
ICU LOS (days)	9.03 ± 3.74	5.13 ± 1.83	5.5 ± 1.91	<0.001^*∗∗*^	<0.001^*∗∗*^	<0.001^*∗∗*^	0.592 NS

One-way ANOVA tests quantitative data between the three groups or more. *P* value, comparison between all group. P1, comparison between IV vasopressors and midodrine. P2, comparison between IV vasopressors and minirin. P3, comparison between midodrine and minirin. ^*∗∗*^Significant level at *P* value < 0.01. Ns, not significant.

**Table 2:** Comparison between the studied groups regarding hemodynamic parameters and laboratory findings (*n* = 90).

	Control	Minirin	Midodrine	*P* value
	Mean ± SD	Mean ± SD	Mean ± SD	
Temperature				
1^st^ day of the study	37.23 ± 0.39	37.2 ± 0.4	37.2 ± 0.62	0.953
Midperiod of the study	37.31 ± 0.35	37.34 ± 0.5	37.11 ± 0.39	0.068
Last day of the study	37.17 ± 0.25	37.14 ± 0.27	37.23 ± 0.4	0.541

Heart rate (HR)				
1^st^ day of the study	120.43 ± 14.64	120.53 ± 11.91	117.5 ± 14.28	0.622
Midperiod of the study	97.1 ± 16.65	102.8 ± 20.77	103.77 ± 16.65	0.311
Last day of the study	96.73 ± 18.75	91.83 ± 12.71	79 ± 16.9	<0.001^*∗∗*^

Mean arterial pressure (MAP)				
1^st^ day of the study	62.87 ± 19.21	65.83 ± 20.29	52.37 ± 11.77	0.010^*∗*^
Midperiod of the study	83.8 ± 14.64	83.9 ± 11.75	77.17 ± 16.63	0.125
Last day of the study	82.57 ± 13.42	82.57 ± 11.94	77.23 ± 15.83	0.231

Respiratory rate (RR)				
1^st^ day of the study	25.33 ± 6.74	23.6 ± 6.93	20.27 ± 5.6	0.011^*∗*^
Midperiod of the study	18.83 ± 4.19	17.53 ± 3.05	17.13 ± 3.27	0.157
Last day of the study	15.7 ± 3.21	15.57 ± 3.11	16 ± 3.13	0.862

Serum creatinine				
1^st^ day of the study	90.35 ± 52.22	99.71 ± 39.97	63.87 ± 34.43	0.005^*∗∗*^
Last day of the study	123.11 ± 112.2	101.95 ± 41.18	92.12 ± 54.76	0.276

Arterial PH				
1^st^ day of the study	7.41 ± 0.09	7.39 ± 0.08	7.41 ± 0.09	0.696
Midperiod of the study	7.42 ± 0.08	7.39 ± 0.08	7.42 ± 0.08	0.268
Last day of the study	7.41 ± 0.07	7.39 ± 0.07	7.41 ± 0.07	0.590

Serum HCO_3_				
1^st^ day of the study	19.91 ± 5.58	20.72 ± 7.22	19.91 ± 5.58	0.842
Midperiod of the study	21.99 ± 3.76	22.06 ± 8.06	21.99 ± 3.76	0.999
Last day of the study	23.14 ± 3.72	23.01 ± 7.05	22.77 ± 3.82	0.959

PO_2_				
1^st^ day of the study	103.37 ± 45.09	102.52 ± 39.24	103.37 ± 45.09	0.996
Midperiod of the study	104.07 ± 30.33	89.53 ± 28.56	104.07 ± 30.33	0.098
Last day of the study	82.49 ± 18.57	83.57 ± 23.24	88.32 ± 25.51	0.582

PCO_2_				
1^st^ day of the study	32.04 ± 9.21	33.5 ± 14.52	32.04 ± 9.21	0.846
Midperiod of the study	30.65 ± 6.6	32.33 ± 14.75	30.65 ± 6.6	0.758
Last day of the study	33.19 ± 4.52	32.87 ± 11.52	31.91 ± 5.3	0.803

One-way ANOVA tests quantitative data between the three groups or more. ^*∗*^Significant level at *P* value < 0.05; ^*∗∗*^significant level at *P* value < 0.01.

**Table 3:** Comparison between the studied groups regarding the total intake of fluid and total output (*n* = 90).

	Control	Minirin	Midodrine	*P* value
	Mean ± SD	Mean ± SD	Mean ± SD	
Total intake of fluid (mL)				
1^st^ day of the study	3243.33 ± 2081.77	2950 ± 1192.87	3753.33 ± 545.66	0.092
Midperiod of the study	2683.33 ± 1122.45	2921.67 ± 1383.57	3976.67 ± 943.92	<0.001^*∗∗*^
Last day of the study	2632.14 ± 1553.56	2828.33 ± 1521.1	4076.67 ± 661.73	<0.001^*∗∗*^

Total output				
1^st^ day of the study	3073.33 ± 2010.91	2366.67 ± 1025.06	3345.33 ± 952.13	0.026^*∗*^
Midperiod of the study	3303.33 ± 1334.94	2514.67 ± 1203.22	3333.33 ± 1466.25	0.031^*∗*^
Last day of the study	3125 ± 1504.9	2527 ± 1587.38	3368.33 ± 1056.14	0.063

One-way ANOVA tests quantitative data between the three groups or more. ^*∗*^Significant level at *P* value < 0.05; ^*∗∗*^significant level at *P* value < 0.01.

## Data Availability

The datasets used and analyzed during the current study are available from the corresponding author on reasonable request.
